# The morphology and attachment of *Protopolystoma xenopodis* (Monogenea: Polystomatidae) infecting the African clawed frog *Xenopus laevis*

**DOI:** 10.1051/parasite/2014020

**Published:** 2014-05-14

**Authors:** Maxine Theunissen, Louwrens Tiedt, Louis H. Du Preez

**Affiliations:** 1 Unit for Environmental Sciences and Management, North-West University Potchefstroom Campus Private Bag X60001 Potchefstroom 2520 South Africa; 2 Electron Microscopy Unit, North-West University Potchefstroom Campus Private Bag X60001 Potchefstroom 2520 South Africa

**Keywords:** Monogenea, Polystomatidae, *Protopolystoma xenopodis*, *Xenopus laevis*, Morphology

## Abstract

The African clawed frog *Xenopus laevis* (Anura: Pipidae) is host to more than 25 parasite genera encompassing most of the parasitic invertebrate groups. *Protopolystoma xenopodis* Price, 1943 (Monogenea: Polystomatidae) is one of two monogeneans infecting *X. laevis*. This study focussed on the external morphology of different developmental stages using scanning electron microscopy, histology and light microscopy. Eggs are released continuously and are washed out when the frog urinates. After successful development, an active swimming oncomiracidium leaves the egg capsule and locates a potential post-metamorphic clawed frog. The oncomiracidium migrates to the kidney where it attaches and starts to feed on blood. The parasite then migrates to the urinary bladder where it reaches maturity. Eggs are fusiform, about 300 μm long, with a smooth surface and are operculated. Oncomiracidia are elongated and cylindrical in shape, with an oval posterior cup-shaped haptor that bears a total of 20 sclerites; 16 marginal hooklets used for attachment to the kidney of the host and two pairs of hamulus primordia. Cilia from the 64 ciliated cells enable the oncomiracidium to swim for up to 24 h when the cilia subsequently curl up, become non-functional and are shed from the body. The tegument between the ciliated cells bears a series of sensory papillae. The body of the mature parasite is elongated and pyriform and possesses an opisthaptor armed with three pairs of suckers and two pairs of falciform hooks to ensure a firm grip on the flexible internal surface of the urinary bladder.

## Abstract (fr)

Le xénope du Cap *Xenopus laevis* (Anura : Pipidae) est l’hôte de plus de 25 genres de parasites représentant la plupart des groupes d’invertébrés parasites. *Protopolystoma xenopodis* Price, 1943 (Monogenea : Polystomatidae) est l’un des deux monogènes infectant *X. laevis*. Cette étude a porté sur la morphologie externe des différents stades de développement du cycle de vie de *P. xenopodis* en utilisant la microscopie électronique à balayage, l’histologie et la microscopie photonique. Les œufs sont libérés de façon continue et sont dispersés quand le xénope urine. Après un développement réussi, un oncomiracidium actif et nageant librement quitte la capsule de l’œuf et localise un potentiel xénope post-métamorphique. L’oncomiracidium migre vers le rein où il s’attache et commence à se nourrir de sang. Le parasite migre ensuite vers la vessie où il atteint sa maturité. Les oeufs sont fusiformes, longs de 300 μm, avec une surface lisse et sont operculés. Les oncomiracidia sont allongés et cylindriques, avec un hapteur postérieur ovale en forme de coupe qui porte un total de 20 sclérites ; 16 petits crochets marginaux utilisés pour se fixer sur le rein de l’hôte et 2 paires de primordia d’hamuli. Des cils portés par 64 cellules ciliées permettent à l’oncomiracidium de nager pendant 24 heures, après quoi les cils se recroquevillent, deviennent non fonctionnels et sont éliminées de la surface du corps. Le tégument entre les cellules ciliées porte une série de papilles sensorielles. Le corps du parasite mûr est allongé et piriforme et porte un opisthohapteur armé de trois paires de ventouses et deux paires de crochets falciformes, assurant une prise ferme sur la surface flexible interne de la vessie urinaire.

## Introduction

The African clawed frog *Xenopus laevis* (Daudin, 1802) [2] (Anura: Pipidae) is primarily aquatic, which allows it to serve as a host or intermediate host for several parasites. *Xenopus laevis* is host to a rich assemblage of more than 25 parasite genera from seven invertebrate groups [33]. These parasites are relatively distinct from those in other anurans, shown by their isolated taxonomic position [33]. Parasites infecting *Xenopus* also benefit from the fact that large numbers of frogs are often confined to relatively small areas of suitable habitat, predominantly when water levels drop during dry seasons [27].

*Protopolystoma xenopodis* (Price, 1943) [22] (Monogenea: Polystomatidae) is one of two monogeneans to infect this anuran host, the other being *Gyrdicotylus gallieni* Vercammen-Grandjean, 1960 [42], that is found in the buccal region of the host. Monogeneans are mainly ectoparasitic on fish, but the family Polystomatidae radiated onto the tetrapods and are found on the skin and gills of the Australian lungfish *Neoceratodus forsteri* (Krefft, 1870) [16], urinary bladder of frogs, gills and skin of salamanders, cloaca and phallodeum of caecileans, on the eye, nostrils, mouth, cloaca or urinary bladder of freshwater turtles and on the eye of the hippopotamus *Hippopotamus amphibius* Linnaeus, 1758 [1, 19, 38]. These host organisms are ecologically related through their association with freshwater habitats that favours parasite transmission. Polystome reproduction is generally limited to the periods when hosts are in water. Strict host specificity as observed in anuran polystomes minimises the chance of host-switching to other amphibian species [12, 38]. Amongst anuran polystomes *P. xenopodis* is probably one of the best studied species with respect to general biology and environmental factors affecting reproduction, transmission and survival [9–11, 35].

Adult *P. xenopodis* are found in the urinary bladder where they continuously produce eggs (mean 9 eggs/worm/day, at 20 °C) [11]. Egg production and output is highly sensitive to temperature fluctuation [10, 11, 39]. Reproduction is continuous, throughout the life of the mature worm, but does fluctuate according to the water temperature [10]. Eggs are expelled into the external environment and infective oncomiracidia hatch in about 22 days. Oncomiracidia swim and actively search for a potential host. Once contact has been established with a potential host the oncomiracidium enters the cloaca and migrates to the kidneys where it develops for approximately 2–3 months. It then migrates back to the urinary bladder where egg production begins 3–4 months postinfection at 22 °C [34, 37, 38]. *Protopolystoma xenopodis* has no uterus and eggs are expelled directly into the host’s urinary bladder [32]. Although cross-fertilisation seems to be the preferred choice amongst polystomes, self-insemination probably occurs via the ovo-vitelline duct in solitary individuals [44]. In comparison with most parasitic platyhelminths, *P. xenopodis* is characterised by low prevalence and intensity in natural populations and low rates of egg production (all reducing output of infective stages into the environment), a relatively long developmental period to egg hatching (delaying transmission) and slow development postinfection to maturity (extending generation time) [38].

This study focussed on the external morphology of *P. xenopodis* using scanning electron microscopy (SEM), histology and clear mount. Egg, larval, subadult and adult stages of *P. xenopodis* will be examined.

## Material and methods

Wild *X. laevis* were caught in July 2013, using baited 20-L bucket traps fitted with an inward directed funnel. Traps were set at various sites in and around the city of Potchefstroom, North-West Province, South-Africa. Permit number 028-NW-11; North-West University ethical clearance: NWU-00005-14-S3. Traps were baited using chicken and/or beef liver, left overnight and retrieved the following morning. To prevent frogs from swallowing the bait, the liver was placed inside a gauze bag which was placed inside the trap. Captured *X. laevis* were individually screened for the release of parasite eggs. Frogs were individually placed in 500 mL plastic tubs containing approximately 250 mL water and maintained at room temperature. After 24 h, the frogs were transferred into clean water and the residual suspended debris was allowed to settle. The supernatant was progressively decanted and the remaining volume was screened using a stereo microscope. A gentle rotating action was used through centripetal force to concentrate sediment particles and eggs in the centre of the tub. The presence of characteristic golden, shiny, fusiform eggs would indicate a positive infection. Tubs with infected hosts were marked. Eggs, larval and adult stages of the life cycle were collected and subsequently prepared for microscopy. Eggs earmarked for incubation were transferred to 6 cm diameter Petri dishes containing dechlorinated water and incubated at 24 °C. The incubation period for *P. xenopodis* was approximately between 22 and 25 days. Development of eggs was monitored using a dissecting microscope, and when fully formed oncomiracidia were observed moving within the eggs, the Petri dishes containing these eggs were placed in direct sunlight for approximately 30 s, resulting in rapid hatching. Hatched oncomiracidia were studied live before they were fixed. Fully embryonated eggs at the point of hatching together with some empty egg shells were collected and fixed in 70% ethanol.

Hosts were euthanised by placing them in a 3% ethyl-4-aminobenzoate (MS 222) solution (Sandoz) for approximately 15 min before they were dissected. The urinary bladder and kidneys were inspected for the presence of parasites. The dark colour as a result of the blood pigment haematin in the gut channel makes it easy to spot *P. xenopodis* within the transparent bladder, but it is less easily spotted within the kidney. The kidneys were flattened between two glass slides and parasites identified by their dark intestines and movement in the kidney tissue. Four positively infected kidneys were fixed in Bouin’s fixative for histological sectioning. Parasites were removed through microdissection; however, it was difficult to remove specimens without damaging them. The bladder was carefully removed and placed in a Petri dish containing a 0.03% saline solution after which it was cut open and parasites were removed and fixed under coverslip pressure either in 10% neutral buffered formalin for light microscopy or in Todd’s fixative for SEM [40]. To fix parasites still attached to the bladder wall, a piece of thin cotton thread was used to tie off the bladder and Todd’s fixative was then carefully injected into the bladder using a 1-mL syringe.

In order to study sclerites of oncomiracidia, 10 specimens were mounted temporarily in lactophenol to clear the specimens. Coverslips were secured using clear nail varnish. Marginal hooklets were studied using a Nikon E800 compound microscope and measurements taken using Nikon NIS Elements software.

For histological sectioning, material was fixed in Bouin’s fixative and subsequently transferred through a graded series of ethanol in 10–15-min steps starting at 30%, then 50% and stored in 70% ethanol. For sectioning, material was further dehydrated in an ethanol series of 70%, 80%, 90% and twice in 100% for 10–15 min each. Dehydrated material was cleared in xylene-ethanol mixture for 10 min and finally in two changes of pure xylene for 20 min each. Material was impregnated with paraffin wax at 60 °C for 24 h; impregnated material was embedded in paraffin wax with a melting point of 65 °C in a Histocene embedding machine. Material was sectioned at 5 μm on a Reichert Yung motorised microtome. Wax sections were placed on a glass slide covered with an albumin adhesive solution, stretched on a stretching plate and dried overnight at 40 °C in an oven. Sections were stained in routine Harris’ haematoxylin and eosin and mounted using Entellan.

For scanning electron microscopy, specimens fixed in Todd’s were washed three times in 0.05 M cacodylate buffer for 15 min each and then washed three times in distilled water for 15 min each. Samples were then dehydrated in an ethanol series; 70%, 90%, 100% and 100% for 15 min each. The samples were critical-point-dried (CPD), mounted on aluminium stubs with the use of double-sided carbon tape, sputter-coated with gold palladium and examined with a FBI ESEM Quanta 200 scanning electron microscope.

## Results

### Egg morphology (Fig. 1)

**Figure 1.**
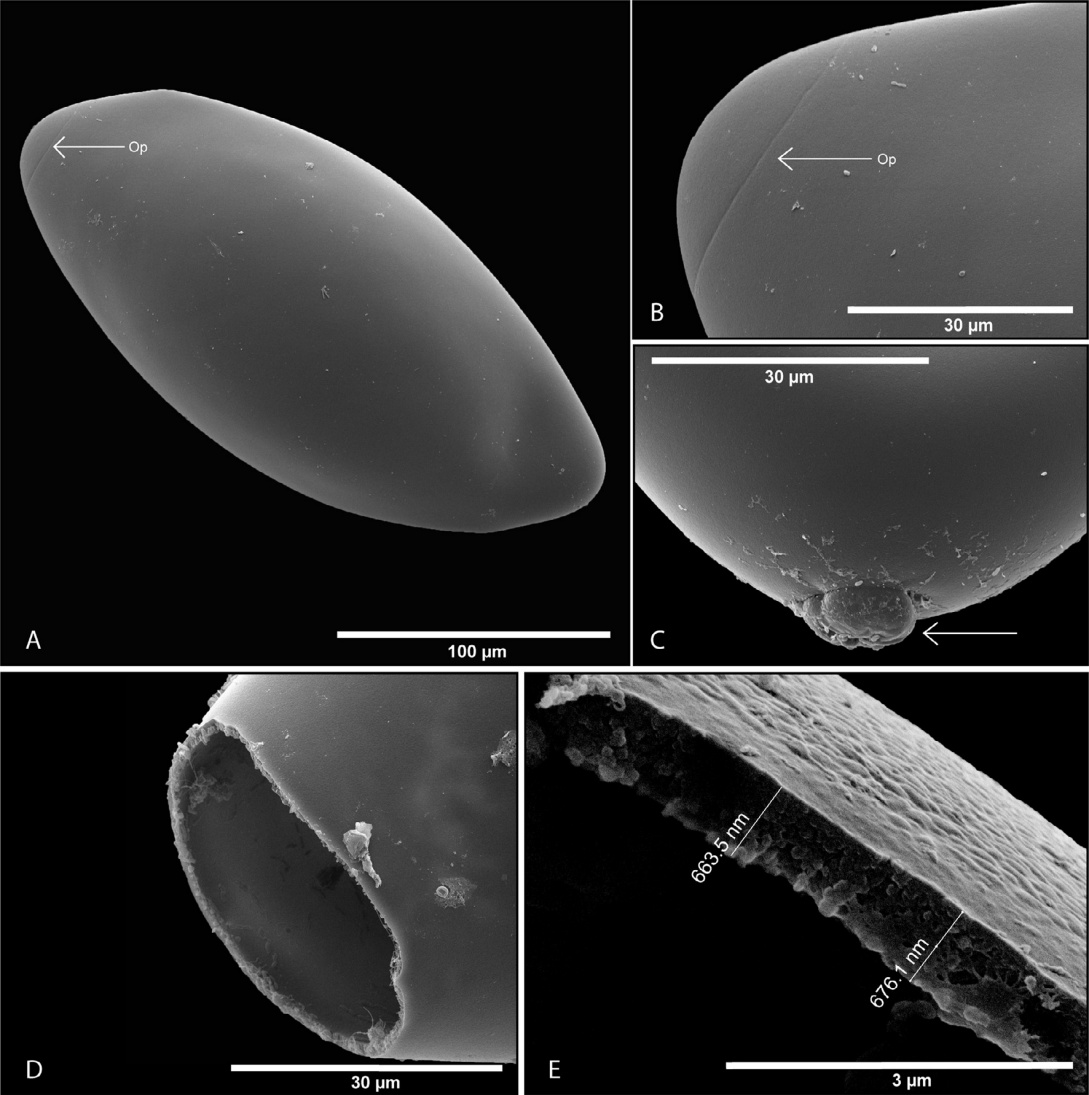
Scanning electron micrographs of *Protopolystoma xenopodis* egg features. (A) Fully embryonated egg, operculum visible (← Op). (B) Operculum becoming visible as the egg develops (← Op). (C) A residual structure on the non-opercular side of the egg. (D) An empty egg shell after the oncomiracidium has left. (E) Egg shell indicating the thickness of an individual parasite egg shell at the opercular opening.

Eggs of *P. xenopodis* are fusiform and operculated (Fig. 1A). The surface is smooth with no visible appendages or filaments attached. In unembryonated eggs, the operculum line is not visible but in incubated eggs, a clear groove where the egg capsule will break open can be observed (Fig. 1B). At the apex of the non-opercular end of the egg, a small residual structure is sometimes noticeable (Fig. 1C). This residual structure was never observed at the operculated side of the egg. Examination of empty egg shells (Fig. 1D) revealed that the operculum breaks off completely with no indication of a hinge structure to keep the operculum attached. Five eggs measured using a scanning electron microscope were 240.9 (230.9–252.1) μm in length and 103.3 (86.8–113.6) μm in width. The egg wall of a single egg measured was 670 nm thick (Fig. 1E).

### Oncomiracidium (Fig. 2)

**Figure 2.**
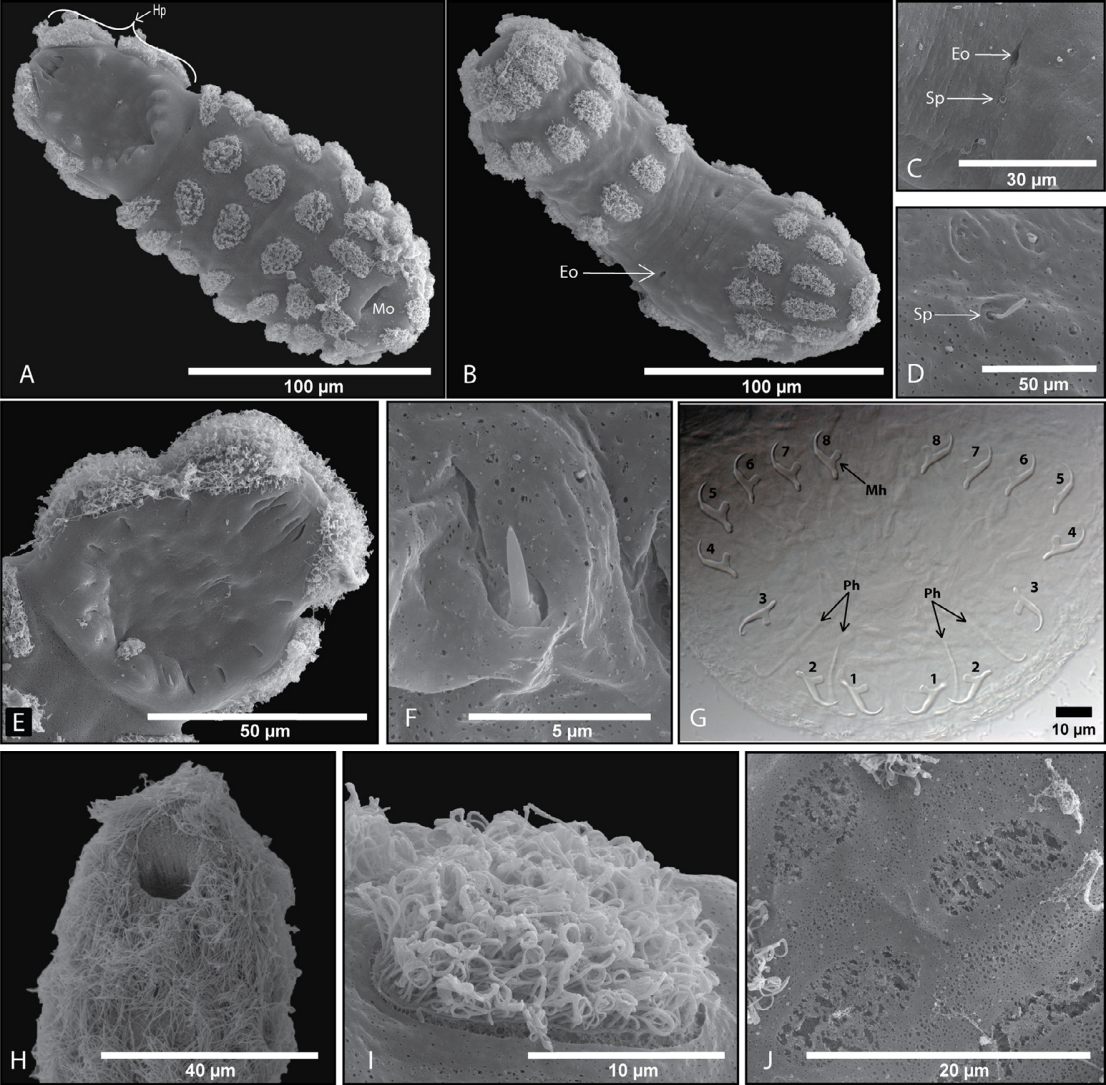
Scanning electron micrographs and one light micrograph (G) of the *Protopolystoma xenopodis* oncomiracidium. (A) Ventral view with posterior haptor (Hp) and anteriorly placed mouth opening (Mo). (B) Dorsal view with two excretory openings in the centre (← Eo). (C) Dorsal view with sensory papillae (← Sp) next to excretory opening. (D) Sensory papillae (← Sp) situated all over the body surface of the parasite. (E) Haptor with 16 retracted marginal hooklets and two pairs of large primordial hamuli. (F) Emerging marginal hooklet. (G) Light micrograph of a lactophenol cleared haptor showing the 20 sclerites; two pairs of eight (1–8) marginal hooklets (Mh) and two pairs of primordial hamuli (Ph). (H) Anterior side of the oncomiracidium covered with cilia. (I) A single ciliated cell with cilia coiling up before cells are shed. (J) Scars after intact cells were shed at the end of the swimming phase.

The body of the oncomiracidium is elongated and cylindrical (Figs. 2A and 2B), 199.3 (193.8–202.6) μm in length, measured from four specimens, using a scanning electron microscope. The mouth is ventral and subterminal. The haptor is directed ventrally, concave and elongated longitudinally (Fig. 2E). Its length is nearly one third of the total body length (Fig. 2A). The haptor bears a total of 20 sclerites. There are 16 marginal hooklets (Fig. 2G), each approximately 13–14 μm, arranged in a circle alongside the primordial of the four hamuli (Fig. 2G). Primordial hamuli protrude posteriorly from the centre of the haptor with a total length of approximately 28–30 μm. The second smaller pair of hamuli lies between the posterior and posterior lateral hooklets (hooklets 1 and 2); these small hamuli are thin, gracile structures, approximately 19–20 μm long. The sclerites are mostly withdrawn (Fig. 2E) when the oncomiracidium is not attached to its host; however, they may sometimes protrude, as shown in Figure 2F. The oncomiracidium is covered in 64 isolated ciliated cells arranged in a symmetrical pattern. Oncomiracidia will actively swim for up to 24 h. In actively swimming oncomiracidia, the cilia are long and appear as a continuous carpet of cilia over the oncomiracidium (Fig. 2H), making it difficult to identify individual ciliated cells. Oncomiracidia that do not find a host in time will lose their ability to swim and become moribund. SEM examination of these moribund oncomiracidia revealed that the cilia curl up and became dysfunctional (Figs. 2A, 2B and 2I). Entire cells are shed, leaving markings on the body (Fig. 2J). The tegument between the ciliated cells bears a series of sensillae (Figs. 2C and 2D); these inhabit relatively constant positions with regard to the ciliated cells.

### Subadult – kidney stage (Fig. 3)

**Figure 3.**
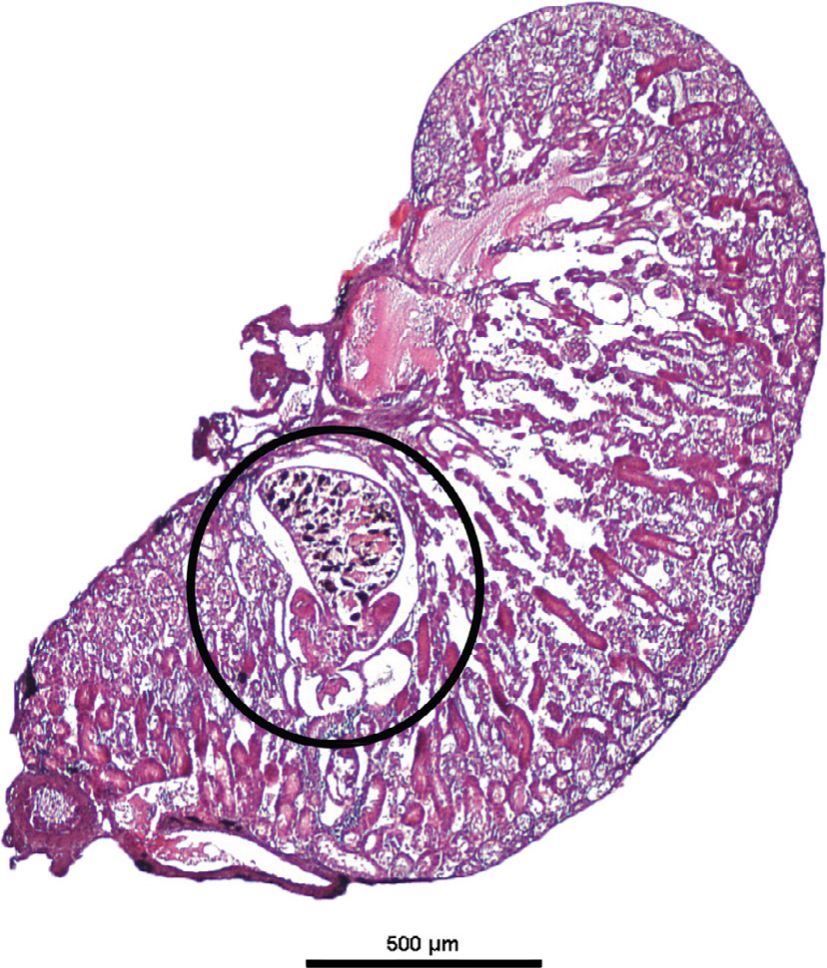
Histological section through a *Protopolystoma xenopodis* within the kidney of a *Xenopus laevis* specimen. The position of the parasite is indicated in the figure.

The oncomiracidia locate a potential frog host and enter the body through the cloaca. Oncomiracidia migrate through the urinary duct to the kidney. The immature parasite attaches inside the kidney (Fig. 3) using its 16 marginal hooklets and most likely the two pairs of developing hamuli. They start feeding on blood and develop in the kidneys. As the parasite matures, it develops six suckers which replace the marginal hooklets as primary attachment organs. The development of suckers was not documented in the present study. The black pigment in the parasite’s gut is through the accumulation of haematin, indicating a sanguinivorous diet [28]. Parasites were observed migrating down the urinary duct towards the bladder where they continue to develop and reach maturity.

### Adult parasite – bladder stage (Figs. 4 and 5)

**Figure 4.**
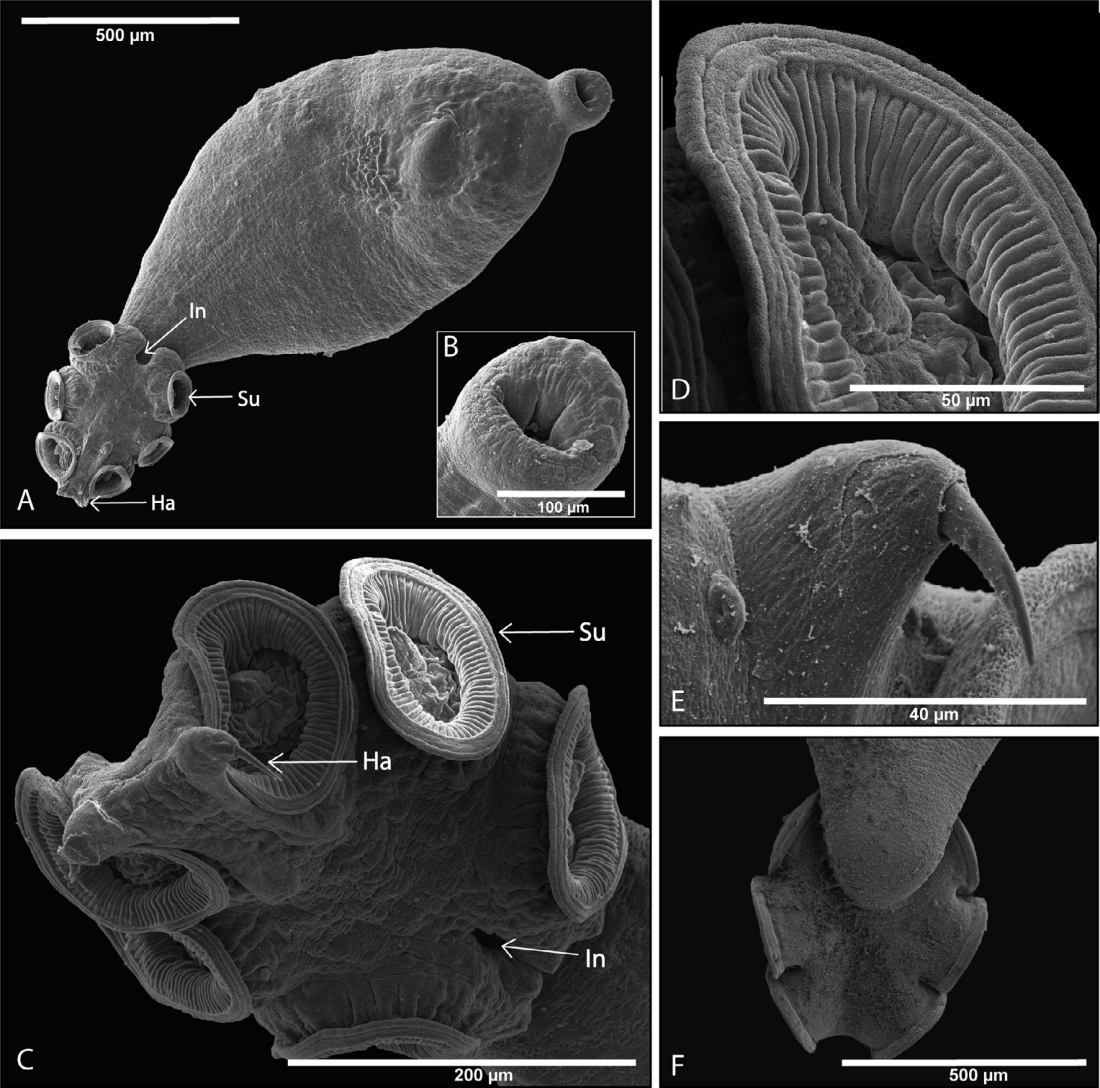
Scanning electron micrographs of mature *Protopolystoma xenopodis* specimens. (A) Ventral view of the parasite showing the mouth opening in the anterior of the parasite and the haptor armed with suckers (← Su), hamuli (← Ha) and a wedge-shaped infolding (← In) between the third sucker pair at the anterior margin of the haptor. (B) Ventral mouth opening. (C) Haptor with six ventral suckers (← Su), hamuli (← Ha) and a wedge-shaped infolding (← In) between the third sucker pair. (D) Single sucker showing the musculature of the sucker. (E) Hamulus point. (F) Dorsal view of the haptor.

**Figure 5.**
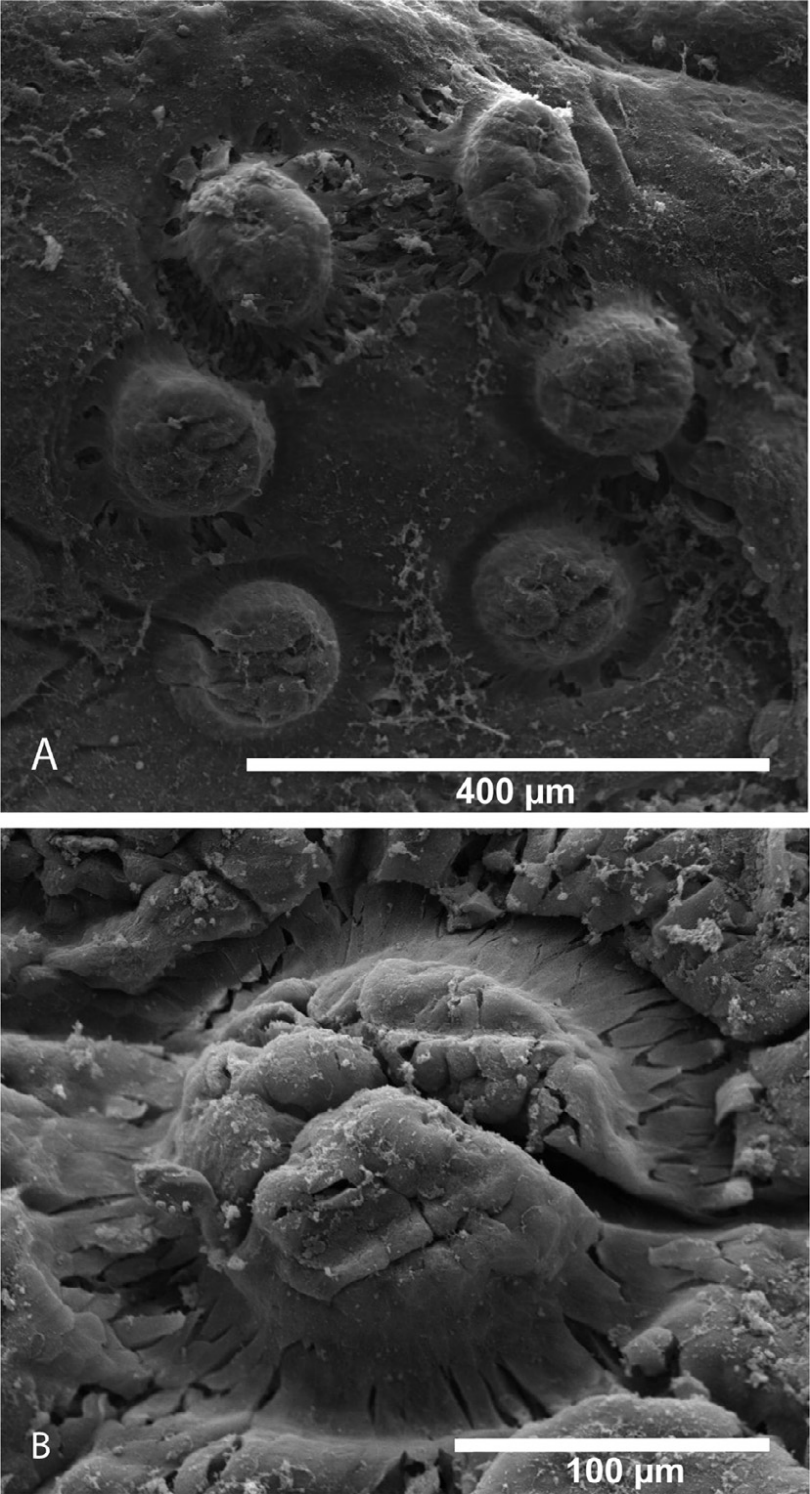
Scanning electron micrographs of buds made by the parasite’s suckers on the host’s bladder surface (A) and high magnification of a single bud (B).

The mouth is subterminal and ventral, with an upper lip protruding over the mouth (Fig. 4B). The body is pyriform, narrowing posteriorly anterior to the haptor. Marginal hooklets are no longer functional but are retained in the body, and attachment to the bladder wall is achieved through six muscular and very flexible suckers (Fig. 4C). Unlike most other polystomes where suckers face ventrally, suckers of *P. xenopodis* face ventro-laterally. Compared with other polystomes the haptor is very flexible, to the extent that whenever a parasite is removed from the bladder and placed in a Petri dish, the haptor folds over and suckers attach readily to the body proper. A wedge-shaped infolding in the anterior margin of the haptor between sucker pair 3 was observed (Figs. 4A and 4C). When attached to the urinary bladder the soft transitional epithelial is drawn into the sucker and when a parasite is removed from the bladder the sucker imprint is clearly visible (Figs. 5A and 5B). Suckers are adapted for attachment to highly contractile substrata such as the urinary bladder.

Attachment is further secured through two pairs of hamuli (Fig. 4E). Only one pair of hamuli develops into large falciform haptoral hamuli, approximately 208 (180–243) μm in length, whilst the second pair of hamuli develops into smaller hamuli, approximately 28 (27–30) μm in length. Suckers are supple and assist in successful attachment to urinary tissue of its host (Figs. 4D and 4F). The body surface is free from ciliated cells or scar tissue as the parasite matures, but still possesses multiple sensillae. Adult parasites in the bladder start producing eggs.

## Discussion

Relative to their body size, the eggs of most monogeneans are quite large [32]. Egg shape varies and, according to Schmidt et al. [24], the shape is determined by the oötype walls. Surface structures and appendages such as filaments are common for monogenean eggs and, according to [41], these structures can be functionally explained in that filaments attach to objects. Egg filaments are absent in polystomes.

Within the Polystomatidae, the shape of the egg varies from oval-round [18, 26], pear-shaped [43], elliptical [5, 21, 43], spindle-shaped [7] to fusiform or diamond-shaped [35] with a smooth surface and no functional appendages. The thickness of the egg shell varies considerably between species. Du Preez et al. (2010) [8] described the egg shell of *Madapolystoma* Du Preez, 2010 [8] as a thin membrane. *Eupolystoma* Kaw, 1950 [13] likewise has a thin transparent membrane as an egg shell with no operculum [30]. The membrane simply ruptures when the oncomiracidium hatches, leaving a collapsed structure with no discernible shape behind. On the other extreme is the thick egg wall of *Oculotrema* with a reported thickness of 3.3 μm [6]. Between the two extremes, a variety of egg shell thicknesses exists and as a rule, polystome eggs are operculated. The eggs of *P. xenopodis* are operculated and have a wall that is 670 nm thick. The opercula on the eggs of *Polystoma australis* Kok et Wyk, 1986 [15], by comparison, are hinged and remain attached once the oncomiracidia have exited [3]. In the present study, no eggs were found with the operculum still attached to the egg casing after the oncomiracidium hatched [41], however, noted that the operculum sometimes does stay attached but did not report a hinge as in the case of *Polystoma* [3]. The colour of polystome eggs varies from whitish in the case of the thin-shelled types of eggs produced by the species of *Eupolystoma* and *Madapolystoma* to the dark, shiny, golden colour of those produced by *Oculotrema* Stunkard, 1924 [25]. The significance of egg shape and shell thickness needs to be studied from an evolutionary perspective. Where eggs develop and hatch inside the host as in the case of *Eupolystoma*, the egg shell is a thin membrane. Eggs that are expelled and develop outside the protection of the host’s body as in the case of *Polystoma* and *Protopolystoma* have thicker egg shells. Polystomes that live under the eyelids of their hosts as in the case of *Neopolystoma* in turtles and *Oculotrema* in the hippopotamus, have eggs with a very thick egg shell [6] that provides protection.

The number and placement of ciliated cells that were observed in this study were similar to those detailed by Tinsley and Owen [38]. Five groups were documented: (1) apical group – 2 cells anterior; (2) cephalic group – 2 × 14 cells, dorsal and ventral; (3) medio-anterior group – 2 × 3 cells, ventral; (4) medio-posterior group – 2 × 6 cells, dorsal and ventral; and (5) haptoral group – 2 × 8 cells, dorsal and lateral. The shapes of the ciliated cells vary in different regions: medially situated cells are often broadly rounded whilst lateral cells are elongated and ellipsoidal [38]. The locomotory cells of monogeneans may be isolated or contiguous [17]. Tinsley [29, 31] briefly described the ultrastructure of polystomatid ciliated cells. The coiling of cilia of moribund oncomiracidia as observed for *P. xenopodis* in the present study (Fig. 2J) has not been reported for polystome larvae. In the present study we observed that when cilia coil up, oncomiracidia lose their ability to swim, and one can assume that oncomiracidia that have not located a host by the time cilia start to lose their functionality will be doomed. Du Preez and Kok's [4], a study on *Polystoma australis* Kok and Van Wyk, 1986 [15] found that cilia are shed, leaving naked cells. It would thus appear that different mechanisms may be at work. The oncomiracidia of some monogenean species shed their ciliary cells immediately after making contact with the host [4, 14, 15, 20], whilst others do not [36]. There seems to be agreement that entire ciliated cells are shed after larvae are established on their hosts. According to Tinsley [31] “the intact cells are thrown off the body surface” of polystomatid oncomiracidia following successful infection.

The mature *P. xenopodis* has a reported lifespan of about 2.5 years [32]. The body surface is smooth, and contains multiple sensillae. Sensory papillae are spread across the surface area of the oncomiracidium, enabling it to navigate and identify potential hosts. Two types of sensillae may be distinguished: small circular “buttons” with a relatively thin encircling wall which are distributed over the surface of the body in no discernible symmetrical configuration, and larger, ellipsoidal, thick-walled sensillae which occur in four regions in *P. xenopodis* (normally three pairs lateral to the mouth, three pairs between and behind the eyes dorsally, two pairs at the junction of the body with the haptor ventrally and several pairs on the posterior lip of the haptor) [38]. Compared with other polystomes, the haptor is exceptionally flexible and manoeuvrable with suckers orientated ventro-laterally. The wedge-shaped infolding in the anterior margin of the haptor between sucker pair 3 has not been reported for any other polystomes. This infolding contributes to the flexibility of the haptor, expanding the area the suckers can reach. As the parasite matures the functional role of attachment of the 16 marginal hooklets are replaced by the six suckers. The suckers are supple and assist in the successful attachment to urinary tissue of its host. The flexible haptor and suckers resemble those of the neotenic form of *Polystoma* which attach to the branchial filaments of tadpoles. Williams [44] stated that the morphology of *P. xenopodis* was in general essentially similar to the neotenic adult of *Polystoma integerrimum* (Frölich) Rudolphi, 1808 [23].

Since the host is permanently aquatic it undergoes continuous osmotic influx, which results in frequent and regular urination (approximately every 2–3 h) [10]. This implies a frequent change in the volume and thus tension on the bladder wall. The thin highly contractile membrane of the bladder thus undergoes intermittent sudden changes in surface area and thickness, potentially causing the haptor to readily detach if rapid stretching or contracting of the bladder tissue is stimulated [27]. In this sheltered site of infection, successful suctorial attachment mechanisms to a highly contractile substrate are of great adaptive advantage to *P. xenopodis*. The flexibility of the parasite’s haptor and suckers along with the two pairs of hamuli probably play a crucial role in attachment within a frequently changing environment.
